# Family therapy sessions with refugee families; a qualitative study

**DOI:** 10.1186/1752-1505-7-7

**Published:** 2013-03-27

**Authors:** Gunilla Jarkman Björn, Per A Gustafsson, Gunilla Sydsjö, Carina Berterö

**Affiliations:** 1 Child and Adolescent Psychiatric Clinic, University Hospital, Linköping, S 581 83, Sweden; 2 Division of Child and Adolescent Psychiatry, Department of Clinical and Experimental Medicine, Faculty of Health Sciences, Linköping University, Linköping, Sweden; 3 Division of Obstetrics and Gynaecology, Department of Clinical and Experimental Medicine, Faculty of Health Sciences, Linköping University, Linköping, Sweden; 4 Department of Obstetrics and Gynaecology in Linköping County Council of Östergötland, Linköping, Sweden; 5 Division of Nursing Science, Department of Medical and Health Sciences, Faculty of Health Sciences, Linköping University, Linköping , S 581 83, Sweden

**Keywords:** Migration, Refugee children, Family therapy, Qualitative method

## Abstract

**Background:**

Due to the armed conflicts in the Balkans in the 1990s many families escaped to other countries. The main goal of this study was to explore in more detail the complexity of various family members’ experiences and perceptions from their life before the war, during the war and the escape, and during their new life in Sweden. There is insufficient knowledge of refugee families’ perceptions, experiences and needs, and especially of the complexity of family perspectives and family systems. This study focused on three families from Bosnia and Herzegovina who came to Sweden and were granted permanent residence permits. The families had at least one child between 5 and 12 years old.

**Method:**

Family therapy sessions were videotaped and verbatim transcriptions were made. Nine family therapy sessions were analysed using a qualitative method with directed content analysis.

**Results:**

Three main categories and ten subcategories were found - 1. Everyday life at home, with two subcategories: The family, Work and School/preschool; 2. The influence of war on everyday life, with three subcategories: The war, The escape, Reflections; 3. The new life, with five subcategories: Employment, Health, Relatives and friends, Limited future, Transition to the new life.

**Conclusions:**

Health care and social welfare professionals need to find out what kind of lives refugee families have lived before coming to a new country, in order to determine individual needs of support. In this study the families had lived ordinary lives in their country of origin, and after experiencing a war situation they escaped to a new country and started a new life. They had thoughts of a limited future but also hopes of getting jobs and taking care of themselves and their families. When analysing each person’s point of view one must seek an all-embracing picture of a family and its complexity to tie together the family narrative. To offer refugee families meetings with family-oriented professionals to provide the opportunity to create a family narrative is recommended for the health and social welfare sector. Using this knowledge by emphasizing the salutogenic perspectives facilitates support to refugee families and individuals. This kind of support can help refugee families to adapt to a new system of society and recapture a sense of coherence, including all three components that lead to coherence: comprehensibility, manageability and meaningfulness. More studies are needed to further investigate the thoughts, experiences and needs of various refugee families and how refugee receiving societies can give the most effective support.

## Background

Refugee families are affected by different types of stressors before the flight, during the flight, and during the resettlement processes. The effects vary and have different time scales for the parents compared to the children [1]. Refugee children tend to be resilient and resourceful despite the many adversities they face [2]. Most children, particularly younger ones, cope with the separation from their home countries more easily than the parents, and they experience fewer barriers to social network rebuilding [3]. In spite of this, many young refugees experience mental health difficulties. Thus the awareness of society and clinicians concerning relevant risks and protective factors is important [4].

Exposure to severe traumatic events in the refugees’ home country, and the medical and psychological effects of this exposure, are known to critically influence the possibilities for resettlement in a new country [5]. Negative health consequences are especially high when relocation is forced due to severe conflicts in the home country associated with violence and man-made trauma [6].

However, post-migration factors such as language barriers [7], loss of culture and support [8], and a prolonged asylum process [9], have also been found to have a negative impact on psychological well-being.

In one study of war-wounded refugees exposed to severe traumas in their home countries, the results indicated that life circumstances and events related to the present situation, “here and now”, were more important for their well-being and social integration than background factors [10]. A review of 22 studies of refugee children found substantial variation in the definitions used and measurements made of the children’s problems and reported levels of post-traumatic stress disorder ranging from 19 to 54% [11].

Traumas and negative life events may give rise to negative changes in attachment between children and their parents. Culturally appropriate counselling theories and their respective interventions can be helpful in finding treatment options [12]. Psychological problems are frequent in refugee children but over time in exile the extent of these problems is reduced. Traumatic experience before arrival is the most important factor determining the short-term reaction of the children, while stressful life in exile seems to be the most important factor affecting the children’s ability to recover from early traumatisation, according to Montgomery [13], who also points out that the quality of family life seems to be important for both short- and long-term mental health. Robertson and Duckett [14] studied displaced Bosnian mothers’ experiences caring for their children during and immediately after the war (1992–1995) and they concluded that although families need to move forward, they may need to look back, at least from time to time. Weine et al. [15] has concluded that qualitative family research is useful for better understanding of refugee families and in helping them through family-oriented mental health services.

Al-Baldawi [16] points out that it is important to distinguish psychosomatic manifestations due to stress from pathological symptoms developed as a result of psychiatric or somatic diseases, in order to reduce the risk of over- or under-diagnosing the patient’s problems, and to choose the correct treatment to promote better and quicker integration. One qualitative study interviewing refugees about their experiences with the Swedish healthcare system showed that care providers’ conversations about daily life were seen as a sign of commitment, knowledge and professional skill [17]. Another study has shown that second-generation immigrant children did not differ from the non-immigrant children in their own presentation of mental health at the age of 12 [18].

A study of Bosnian war prisoners who came to Sweden points out that the most important factor for their well-being during the first period in exile was whether or not the family and other relatives were reunited and if they knew what had happened to other members of their family [19]. A study examining the functioning of the family and the child’s psychological adaptation while staying in a refugee camp in Sweden concluded that family members should not be separated during the asylum and that a follow-up process is desirable when they have obtained residence permits allowing them to stay [20]. Hopes regarding education and family reunion were central in the resettlement of West African refugees in Sweden [21]. In one study evaluating mental health and social adjustment of Iranian children 3,5 years after arrival in Sweden, the conclusion was that current life circumstances in receiving host countries, such as peer relationships and exposure to bullying, are of equal or greater importance than previous exposure to organised violence [22]. Another study showed that extended family and, in particular, parental siblings play important roles in the acculturation experience and family functioning of Vietnamese refugee families in Norway [23].

Goldin [24] found that protection of the refugee children was associated with, among other things, a warm family climate and above all a family sense of hope for the future. Alinder et al. reported positive effects of family sessions after treatment at home of eight families from Bosnia who had fled to Sweden [25].

Due to the armed conflicts in the Balkan region in the 1990s many families escaped to other countries. During the period 1992–1995, nearly 50 000 persons coming from Bosnia and Herzegovina were granted permanent residence permits to stay in Sweden [26].

Research on refugee family perspectives in order to obtain a fair idea of their complexity is of the utmost importance to create useful guidelines for professionals in the health and social welfare sector. Most studies have focused mainly on individual perspectives. Few studies focusing on the family perspectives of refugees have been carried out. Thus there is a need for more research work to be done in this area. The main goal of this study was to explore in more detail the complexity of various members’ experiences and perceptions from their life before the war, during the war and during their escape, and finally during their new life in Sweden.

## Methods

### Study setting

Refugee families from Bosnia-Herzegovina were asked to participate in this study by a nurse in a medical health centre or by social workers in the communities where the families lived. This study focused on using a qualitative method to study the lives and experiences of three refugee families who came from Bosnia-Herzegovina to Sweden. It is part of another project in which initially 14 families participated [27]. That project was an intervention study with the aim of creating a family narrative and supporting the whole family by giving three family therapy sessions. The diagnostic interviews before the intervention with the family therapy sessions were carried out between 1995 and 2000. The families had arrived to Sweden between 1992 and 1995. Eleven out of 14 families who were initially recruited took part in the family therapy sessions. The inclusion criteria were that the families should: 1) come from Bosnia-Herzegovina, 2) have permanent residence permits in Sweden, and 3) have at least one child between five and twelve years old.

### Study group

Three families who had participated in family therapy sessions were chosen in this part of the study. They were selected because of the rich descriptions shown in the transcripts. The material consists of three sessions from each family i.e. a total of nine family therapy sessions. The families consist of both mother and father, and in two of the families one child, and in the third, two children. The ages of the children were: one aged four, two aged seven, and one aged twelve. The three families had been in Sweden for about two years, four years and six and a half years respectively at the start of the first family session. The children in this study had no pronounced psychiatric problems and to our knowledge their parents had not sought psychiatric help for them.

### Data collection

The families received three sessions of family-based therapy in which the children participated with their parents. Each session lasted about one hour, so in total approximately nine hours of session data was analysed. The purpose of giving the family these sessions and going through different themes illustrating their life before, during, and after the war was to give everyone the possibility of being involved, and of sharing experiences and thoughts with each other, to tie together a family narrative with the aim of supporting the whole family. One idea was to open the communication within the family to help the family members to handle stressful and new situations better. Another idea was to share thoughts and experiences to prevent concealment of destructive family secrets. The intervention was influenced by systemic and narrative approach with crisis and salutogenic theory as the framework [28-30]. The themes in the sessions were: former life situation before the war, the war, the escape from the home country from each family member’s point of view, the present situation in regard to role changes, network, thoughts about the future, and coping strategies in the family. The war was mentioned in passing. In comparison to other themes, less time was spent talking about the war. The intention of the therapists was not to focus on traumatic experiences, but to keep the family story on track. All family members, including children, were involved in talking during the sessions. In this study two well-trained and experienced interpreters were involved. All sessions, except one, used an interpreter (the absence was due to sick leave). The sessions were conducted in Swedish and when needed the interpreter spoke the families’ mother tongue.

All sessions were videotaped. Verbatim transcripts were made from the videotapes. Interactions between the family members were noted. The first author was in charge of the family therapy sessions together with a social worker; both were trained in family therapy. The same structure and approach was kept in all family sessions.

### Analysis

Data were analysed using directed content analysis. Content analysis is a method of analysing verbal or written communication in a systematic way [31]. This method is used to interpret meaning from the content of text data [32]. With a directed approach, analysis starts with an analysis of the different themes that have already been selected and focused on in the family therapy sessions. Material from all three sessions with each of the three families was analysed in this study. Transcripts from each session were read several times. First the text was read so that the different themes could be selected in the family therapy sessions. The next step was to collect the text material belonging to each theme from the nine different family therapy sessions. The text was then re-read several times and meaning units assigned to a single topic were sorted into three categories. The three main categories were subdivided into ten subcategories. The categories and subcategories were then validated through a systematic analysis of the material, and the analysis and findings were checked by an experienced and skilled qualitative researcher (author CB).

### Ethical considerations

The ethical principles of autonomy, non-maleficence, beneficence and justice were considered. Concerning autonomy, the families had agreed to participate in the study and they knew they could end their involvement at any time. One dilemma to consider was that talking about traumatic experiences might remind them about hard times and thus might worsen their current mental condition. On the other hand, there was also a possible gain represented by the principle of beneficence. The purpose was to help the family members to leave traumatic experiences behind and continue their new life in a better way. The work with the family therapy sessions was done without taking account of gender, social or economic status, ethnicity or any other factor. The study was approved by the Ethics committee of the University of Linköping (93092).

## Result

**Figure 1:**
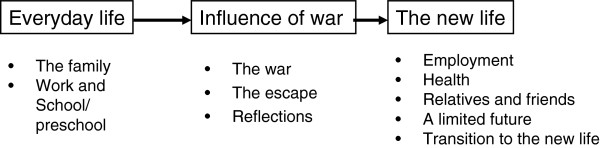
The main categories and subcategories illustrating the time process.

Three main categories emerged from the analysis of the family therapy sessions: “Everyday life at home”, “Influence of war on everyday life”, and “The new life”. A total of ten subcategories comprised the main categories, as shown in Figure 1.

### Everyday life at home

Everyday life at home was the main category that emerged from the analysis of the material from the sessions focusing on life before the war. Narratives came up about family members who still live in Bosnia and Herzegovina and who the informants missed very much. The informants said positive things about relatives who lived with each other and with whom the informants associated often and closely, and who helped them in different ways. Besides family, work and school/preschool were important. The parents did well at their jobs in Bosnia and Herzegovina and their relatives helped them. Family life functioned well economically. The informants had some difficulties at first in remembering what had happened in their home country before the war but they remembered better after they had been talking for a while. Overall, the informants highlighted mostly the positive aspects of life before the war.

#### Family

Family life before the war was elaborated on. Many relatives lived close by and sometimes even lived with the family. Children had access to grandparents and socialized closely with them. Positive traditions with many relatives who celebrated ceremonies together were described. One informant described, for example, birthdays when as many as 20 people often gathered to celebrate together. The informants said that they lived a good life, being able, for example, to go on vacation to the seaside every year. They also mentioned other free time activities like visits to forests.

“*We lived a normal*… *life*” (mother)

#### Work and school/preschool

Work concerned the jobs held by the adults, and school/preschool concerned day-care centres since the children were small when they were in the home country. The parents said that they liked their jobs in their home country and that because they had these jobs and earned money, the family functioned well economically. Getting help and support from family and relatives were common. The parents had access to day-care centres and the children had both positive and negative memories. An example of a negative memory from a parent was remembering when a pre-school teacher at the day-care centre shouted at the children. Some positive memories were having access to day-care centres while the parents were working, and living in close proximity to day-care.

“*Freedom*…*both economically and mentally* …*we had more*…*we worked on both*…” (mother)

### The influence of war on everyday life

This quiet everyday life was suddenly changed when war broke out. Some informants were close to the war zone, while others were further away but were still affected. Ethnic cleansing was mentioned. The informants escaped from their home country in different ways, with part or the whole family together. War, escape and different reflections from this period of war and escape are described under this main category.

#### War

Life was changed dramatically because of the war when the families began to experience bombings and shooting. One was suddenly in the midst of war and saw these events at close range.

“*I saw a bomb fall in the meadow*” (child)

“*A bomb was thrown into a garden*…” (father)

“*I heard bombings in town*” (mother)

One family was not as close to the war zone as the family just quoted above, and they compared themselves with the families who had experienced much worse things than they themselves had.

#### Escape

Escape was expressed as the fleeing to Sweden. In two cases the whole family came together. In the third case one parent and the child came first and the other parent arrived after a few months. One of the families first went to Croatia and got passports there and then came to Sweden. Feelings (crying, fear), somatic complaints (for example vomiting), and depressive symptoms were described. Uncertainty about where to escape to and how to do it were brought up. Family members gave different descriptions depending on their specific experiences.

“*Yes, yes we came together with eleven others*” (father)

“*.…one Sunday when we went, …. we heard on the radio that it could happen that we would be arrested*” (father)

“*Yes, I had to wait there for the aircraft even though it was very windy, when the aircraft came it was so windy as it never have been… eight billion aircrafts there or more…it was very windy. That I remember*” (child)

#### Reflections

The informants presented different reflections. Family members reflected on changes and difficulties associated with the war and the escape. Another change highlighted was the language. One family lived away from the war but had friends who lived close to the war zone. Different stories were told, depending on what traumatic experiences they had been through. Parents in mixed marriages in which the spouses came from different ethnic groups reported difficulties arising from this. Someone said that it was better to forget what had been. One informant reflected about the big change with a negative war situation that later on gave an opportunity to find a new and good life. They mentioned things that were important in their home country but were left behind there, for example toys.

“…*best to forget about what has happened*” (father)

“*We do not only look at one side*…*but at all three sides*” (mother)

### The new life

The third main category was the new life. Different ways to adapt to a new society were described here under the five subcategories related to this main category. The process of finding jobs and learning a new language were mentioned. In their new life some of them were dealing with health conditions linked to the war and to traumatic experiences, but also ordinary health problems such as colds and infections. Much effort was put into keeping the family together and making new friends. Much of the talking was about here and now, with expressions of feelings of a limited future. The informants expressed their thoughts about their transition to a new life.

#### Employment

There were reports that much time was spent on learning Swedish and on job searching and on getting children started with school work. It was not easy for parents to find a similar job to the one they had in their home country. Also, day-care matters played a part in employment difficulties.

“*In the beginning it was difficult because it was kind of a problem for the children to adjust*, *and day*-*care and everything*…” (mother)

Dilemmas in making different aspects of life work together were highlighted, for example, when a person had got a job but had to go there by car and there was a delay starting the job because of the need to get a driving license first.

#### Health

The new life was accompanied by health aspects linked to cultural differences and traumatic experiences, as well as by ordinary health problems that children face, for example repeated colds and infections. War injuries were discussed, and various operations and forms of bodily harm resulting from these were mentioned. Traumatic memories and nightmares were mentioned, and also sleeping problems and depressive symptoms. Hearing disorders were mentioned and different assessments and ways to cope with these were discussed as well.

“*…she is sitting at the table (the daughter) eating and cannot breathe,.one or two spoonfuls of food and cannot breathe through her nose..does not manage to eat*” (mother)

“*Well, on the whole, still affected by it, I mean that I sleep badly*” (mother)

“*You get tired*” (father)

#### Relatives and friends

Concerning the subcategory relatives and friends, the importance of having relatives was mentioned and some stories were told about relatives being separated and living in different countries around the world. In one case, a couple attempted to bring in parents from their home country to stay with them in Sweden. Visits of grandparents were described in detail as well as natural deaths and how these had influenced the family. One informant talked about having no real friends in Sweden. Difficulties in socializing and making friends were described.

“*Not as much contact with Swedish children*…*as there could be*” (father)

#### Limited future

The subcategory of the limited future was named so as to capture the difficulties the grown up informants described in thinking far ahead about the future. Many of their tales were about the situation here and now. It also appeared that not all difficulties were linked to cultural differences between living in different countries, but instead that persons had different personalities and that experiences, at least to some extent, depended more on this than cultural differences. The children could see a future which was not limited.

“*I cannot think five years ahead*” (father)

“*We do not talk much about the future*” (mother)

“*I want to be a doctor or work at a pharmacy. I change often, I do not know…*” (child)

#### Transition to the new life

Concerning the transition to the new life, there was a positive attitude towards getting permanent permission to stay and thus being able to avoid having to move around with suitcases.

“*What is important for us* …*is to work*…” (father)

One person emphasized the importance of complying with Swedish laws and regulations. Several of the families had broken up several times in Sweden and lived in different refugee camps. The informants talked about couples having different roles in their home country but also in their new life. They did not focus on gender differences but more on differences between living in the countryside and in town. One informant spoke, for example, about a grandfather who was a better cook than anyone else. Thoughts of not getting stuck in the past came up.

“*You have to move on and not think about what happened before*” (mother)

“*We used to put emphasis on the future of the children*” (father)

### Evaluation of the sessions

At a follow-up interview with parents about one year after the last family therapy sessions the responses to these sessions were positive. The parents reported that their children felt better. Some doubts were expressed by one of the parents in one family about talking with children about traumatic experiences. This parent said that it would be better for the children if they forgot about what had happened.

## Discussion

This study examined life and experiences of three families from Bosnia and Herzegovina who had fled from the war and got permanent residence permits to stay in Sweden. The families said that in the Balkans they had lived a life which they described as normal and good most of the time. Life was changed because of the war situation and they were forced to start all over again. The parents’ thoughts about the years ahead can be interpreted to mean that they feel they have limited scope in the future. On questions about life within five years they had problems thinking that far ahead. Much focus was instead on more narrow problems such as getting jobs and taking care of themselves economically. The children did not have worries or problems when talking about jobs. For them it seemed more certain that they would get jobs in the future. In the attempt to generalize the findings from qualitative studies, as in all research, it is necessary to consider if the findings, based on the data presented, are transferable to other similar groups [33]. Salutogenesis is important theoretically when meeting these families. Antonovsky [30] developed the term “salutogenesis”, and the main concept in his theories is a sense of coherence. The need for coherence gives an explanation for the role of stress in human functioning. The sense of coherence has three components: comprehensibility, manageability and meaningfulness. These components seemed valuable when interpreting what the family members were talking about in the sessions. The intention to not focus on traumatic experiences probably influenced the time used to talk about war and other traumatic experiences. Another explanation could be that the family members did not want to be reminded about the war and handled the situation by neglecting that issue. It seemed important for the families who had experienced a concept of coherence in their home country also to have a sense of coherence in the new country. Their statements, for example, about having close relations to family and relatives, and their job situation in their home country showed the importance of coherence. The war interrupted their sense of coherence. Several of the members stressed the importance of making friends and getting jobs in the new country and in that way getting a new sense of coherence.

Bronfenbrenner’s ecological theory of human development [34] is another theory that is worth considering when trying to understand refugee families in their new life. He analysed different types of systems that aid in human development. To understand a child’s development and situation it is important to not only look at the child, its family, and its immediate environment but also at the interaction with the wider environment. The families in this study talked about their new life where not only the immediate environment influenced them but also the interaction with the wider environment, such as how society is built up and what kind of culture, rules and regulations there are in the new country. For the refugee children, the support, not only of their families, but also of school teachers and their new friends, were of equal importance. Also, their contacts with their extended family, perhaps living in another part of the world, were described as important.

The main categories found in this study seem reliable, but could possibly be different in other refugee groups. The subcategories would probably differ more, particularly if the families came from another cultural background. These families had a good life before the war and before the flight to Sweden, with no psychiatric problems. Because of the traumatic events which they have gone through they might be vulnerable. Even if the children do not have psychiatric problems it is valuable to get information from the child itself in order to understand the child’s psychological condition [27]. The UN convention on the Rights of the Child [35] is important, among other things, for how it impacts the interplay between government policy and practice and refugee children’s welfare [36]. Refugee children constitute a vulnerable group, which is in need of special care and attention [37]; however few studies to date have focused on the assimilation in the new country viewed from a longer time perspective [38].

The intention of this study was to focus on and listen to all members of the family, but less space was allocated to children’s statements. One explanation could be that it was easier for the grown-ups to remember things about their home country than it was for the children, and the therapist continued to ask more questions about their memories. One of the children was less than one year old when arriving in Sweden and therefore could not express memories from the home country. Another explanation could be the tradition that grown-ups talk more with each other than with children.

The doubts expressed by a parent in one family during the follow-up session to our study about talking with children about their experiences could illustrate a known phenomenon that has been described by Almqvist & Broberg [39] as a strategy of denial and silence within a family about previous traumatic experiences. This strategy of mutual silence might become an obstacle for giving traumatised children parental support and professional treatment.

In research as well as in therapy sessions, basic ethical principles such as autonomy, non-maleficence, beneficence and justice should each be taken into consideration. These principles may be considered from the point of view of each of the actors involved: the patient, the family members, the therapist and the interpreter [40].

When using an interpreter, potential threats to validity arise at various points [41]. Methodological issues with respect to interpreters have received only limited attention in cross-cultural interview studies [42]. An interpreter provides verbal translation during an interaction/conversation between two or more persons who speak different languages. The quality of data and translation of speech can affect the accuracy of any study. One must pay attention to the risk that the person interpreting could modify participants’ responses to what she/he thinks the clinician or researcher wants to hear. Interpreters are active but neutral during the data generating process. In this study the person leading the sessions (GJB) did not understand the mother tongue spoken by the family members and the interpreter, so it was not possible to control any bias in the interpreting. In Sweden there is a law [43] stating that people who do not understand or speak Swedish have the right to an interpreter in all contacts with public authorities. Interpreter agencies supply well-educated and authorized interpreters [44]. They are educated in language, laws and regulations, secrecy, professional attitudes and medical terminology. In this study, to minimize possible bias, well-trained and experienced interpreters employed by an interpreter agency were selected. The interpreters were known to have broad experience as interpreters in clinical work and were recognised as professionally competent. In a general sense there is a need to develop and evaluate specific and appropriate training programs for interpreters as well as for clinicians working in child mental health, as has been pointed out by Rousseau et al. [45].

Several limitations of this study have to be taken into consideration. One is that only a few cases are examined. Another limitation is that the families came from one part of Europe and the adults were well educated and had lived a life quite similar to other European people and might not be comparable with other refugee groups from other cultures. There were some technical problems in the video-taping. Some words and sentences could not be interpreted in spite of reviewing and listening several times and in spite of another person watching and listening to the taping. Other limitations are the therapists’ lack of linguistic skills and how interviews might be affected by the presence of an interpreter [41]. In all the sessions except one there was an interpreter present, and it is not known how accurate the interpretation was. In some sessions the interpreter was attending but was silent throughout the session since Swedish was spoken by all members of the family. One has to consider whether the interpreter had an effect on the communication, for example if family members would have said other things if an interpreter had not been involved. In order to limit the risks identified and reduce misunderstandings the intention was to use the same interpreter in each family for all three sessions. For two out of three families the same interpreter was involved in all three sessions. All families had members who knew Swedish fairly well and were able to evaluate the interpreter’s translation as accurate.

## Conclusions

In order to determine what the individual needs of support are in refugee families it is valuable to find out what kind of lives these families have led before coming to a new country. In this study the families had lived normal lives in their country of origin, similar to others around them, but after experiencing a war situation their lives changed when they escaped to a new country and started a new life. Even if they had thoughts of a limited future they had hopes of getting jobs and taking care of themselves and their families. It is important to get an all-embracing picture of a family and listen to each person’s point of view to understand the complexity of the family system and tie together the family narrative. A recommendation for the health and social welfare sector is to offer refugee families with children meetings with professionals who have family-oriented knowledge. The purpose would be to let the family members tell their individual experiences while the others are listening, so that all can be joined into a family narrative. If someone has psychiatric problems, for example, depression, it is important to also offer individual treatment. Family therapy can be helpful in strengthening the family members’ ability to cope with life by providing a common picture of the complexity of the family system but it is not enough to cure individual psychiatric problems. Some limitations of family therapy interventions could be cultural aspects, for example, the idea that grown-ups are the ones who should talk, with the consequence that the children do not speak much. There may be views that children need to be protected against talking about traumatic experiences. The complexity of family perspectives and family systems is important to consider when handling psycho-social support to refugees. In family therapy it is known that if one individual does not feel happy, it will influence the whole family. There is a risk that this phenomenon will be magnified in refugee situations, since the families’ normal social networks have usually deteriorated. If everyone is heard you get to know different thoughts and feelings and with support it is possible to help the family on the whole to feel better. More focus and space could be given to the children to add to the knowledge of the complexity of the family. Using knowledge by emphasizing the salutogenic perspectives facilitates the provision of support to refugee families. This support helps refugee families to adapt to a new system of society and recapture a sense of coherence, including all three components: comprehensibility, manageability and meaningfulness. More studies are needed to further investigate the perceptions, experiences and needs of various refugee families, and especially the complexity of family perspectives and family systems.

## Consent

Informed consent was obtained from the families for the whole research process.

## Competing interests

The authors declare that they have no competing interests.

## Authors’ contributions

GJB participated in the design of the study, carried out the data collection, participated in the analyses and drafted the manuscript. PG participated in the design of the study, the discussions of the analyses, and helped to draft the manuscript. GS participated in the discussions of the analyses and helped to draft the manuscript, and CB participated in the design of the study, in the analyses and helped to draft the manuscript. All authors read and approved the final manuscript.
